# Macrophage Biology and Mechanisms of Immune Suppression in Breast Cancer

**DOI:** 10.3389/fimmu.2021.643771

**Published:** 2021-04-23

**Authors:** Anita K. Mehta, Sapana Kadel, Madeline G. Townsend, Madisson Oliwa, Jennifer L. Guerriero

**Affiliations:** ^1^Breast Tumor Immunology Laboratory, Dana-Farber Cancer Institute, Boston, MA, United States; ^2^Division of Breast Surgery, Department of Surgery, Brigham and Women's Hospital, Boston, MA, United States

**Keywords:** breast cancer, tumor associated macrophages, immune suppression, T cell inhibition, cancer immunity

## Abstract

Macrophages are crucial innate immune cells that maintain tissue homeostasis and defend against pathogens; however, their infiltration into tumors has been associated with adverse outcomes. Tumor-associated macrophages (TAMs) represent a significant component of the inflammatory infiltrate in breast tumors, and extensive infiltration of TAMs has been linked to poor prognosis in breast cancer. Here, we detail how TAMs impede a productive tumor immunity cycle by limiting antigen presentation and reducing activation of cytotoxic T lymphocytes (CTLs) while simultaneously supporting tumor cell survival, angiogenesis, and metastasis. There is an urgent need to overcome TAM-mediated immune suppression for durable anti-tumor immunity in breast cancer. To date, failure to fully characterize TAM biology and classify multiple subsets has hindered advancement in therapeutic targeting. In this regard, the complexity of TAMs has recently taken center stage owing to their subset diversity and tightly regulated molecular and metabolic phenotypes. In this review, we reveal major gaps in our knowledge of the functional and phenotypic characterization of TAM subsets associated with breast cancer, before and after treatment. Future work to characterize TAM subsets, location, and crosstalk with neighboring cells will be critical to counteract TAM pro-tumor functions and to identify novel TAM-modulating strategies and combinations that are likely to enhance current therapies and overcome chemo- and immuno-therapy resistance.

## Introduction

**Figure 1:**
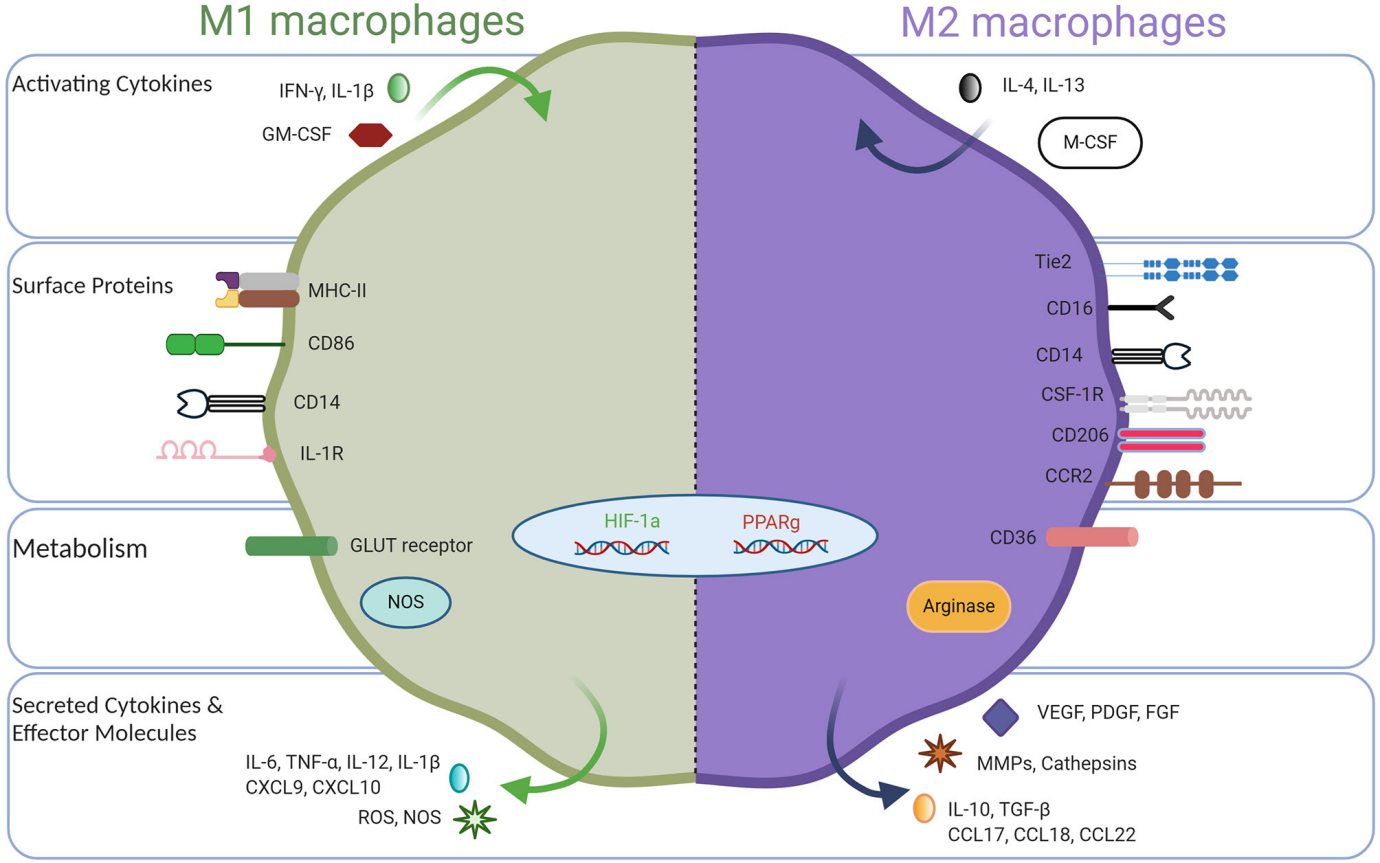
Opposing phenotypes of M1-like and M2-like tumor-associated macrophages.

Macrophages are innate immune cells and play a myriad of important roles such as host defense, tissue homeostasis, and modulating inflammatory responses (1, 2). To perform these functions, immature macrophages with high plasticity respond to microenvironmental cues, causing them to adopt a spectrum of effector function, among which M1-like and M2-like represent extreme polarization states (3–5). Classically activated M1 macrophages exhibit pro-inflammatory behavior by migrating to inflamed tissues, targeting pathogens with the production of reactive oxygen species (ROS), and having high antigen-expressing potential (6–8). Due to their inflammatory behavior, anti-tumor macrophages are commonly called M1 macrophages. These macrophages can be potent effector cells that kill tumor cells and can recruit cytotoxic T lymphocytes (CTLs) to activate adaptive immune responses. On the opposite side of the macrophage polarization spectrum, alternatively activated M2 macrophages secrete anti-inflammatory cytokines to induce immune tolerance and attract T regulatory cells (Tregs) and Th2 T cell subsets capable of protective type 2 responses but devoid of cytotoxic functions. M2 macrophages facilitate canonical tissue repair functions and in cancer are regarded as pro-tumor where they promote tissue remodeling and repair, stimulate angiogenesis with vascular endothelial growth factor (VEGF), and encourage tissue growth with transforming growth factor beta (TGF-β) (9). Therefore, for simplicity, tumor-associated macrophages (TAMs) have been described as either M1-like (anti-tumor) or M2-like (pro-tumor), but it should be recognized that the M1/M2 dichotomy represents idealized polarization states (Figure 1), while in nature, there exists a broad spectrum of macrophage phenotypes, which will be discussed in detail below.

## Tumor Infiltrating Macrophages in Breast Tumors

### Recruitment of Monocytes and Macrophages to Breast Tumors

**Figure 2:**
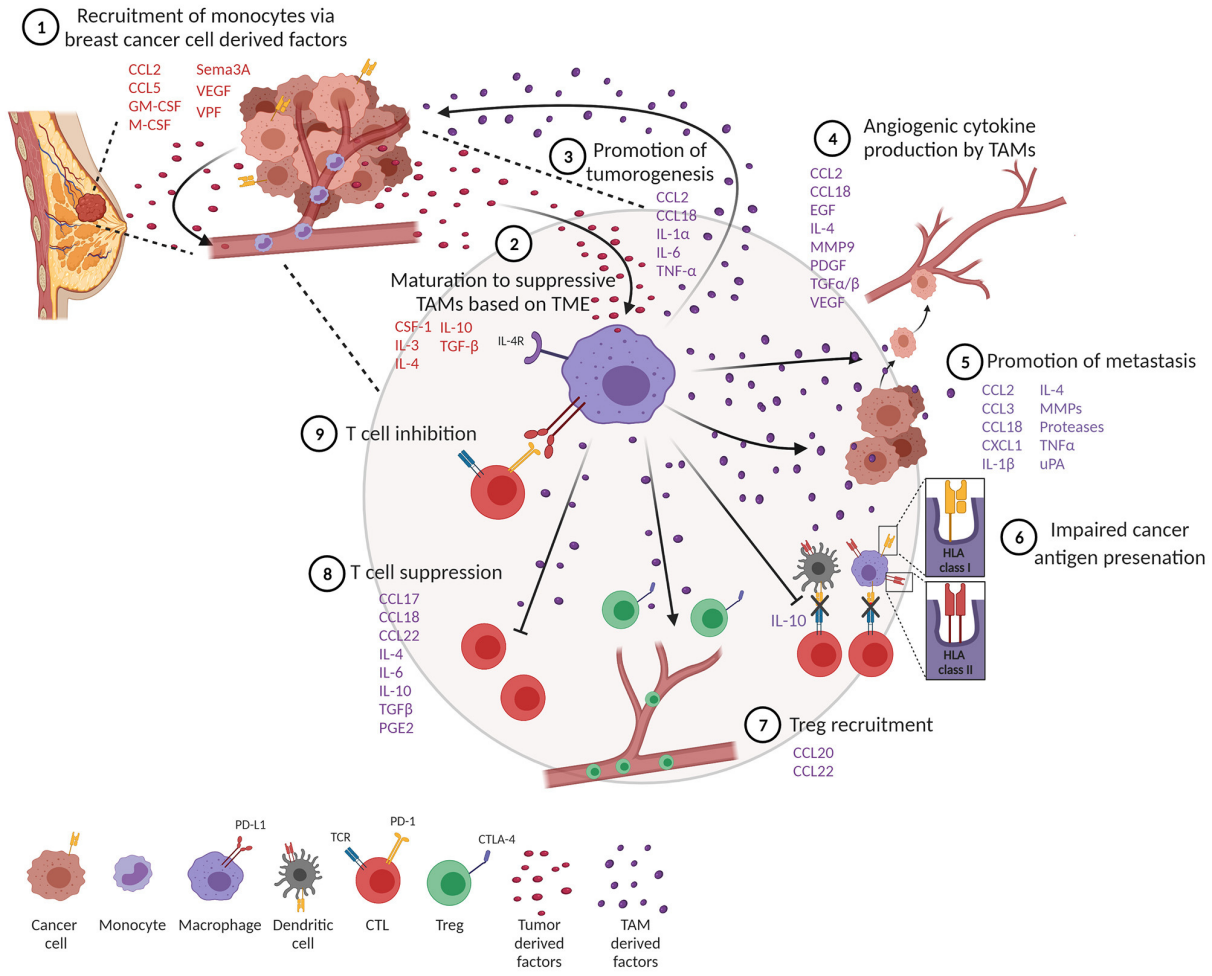
Tumor-associated macrophages (TAMs) impede a productive anti-tumor immune response. (1) Monocytes are recruited to the tumor through tumor-derived factors. (2) One of the tumor monocytes mature to suppressive TAMs. (3) TAMs produce factors that promote tumor cell proliferation and survival. (4) TAMs promote angiogenesis and metastasis (5). (6) TAMs impair productive antigen presentation by dendritic cells and themselves downregulate MHC class I and II molecules. TAMs inhibit T cell function through recruitment of T regulatory cells (Tregs) (7) and suppression (8) and upregulation of co-inhibitory molecules (9).

TAMs represent a significant component of the inflammatory infiltrate in breast tumors (10, 11). Tumor-derived growth factors such as chemokines and cytokines facilitate recruitment of monocytes and macrophages into tumors (Figure 2, step 1) (12). One of the best-characterized cytokines responsible for recruiting TAMs into the tumor is chemokine (C-C motif) ligand 2 (CCL2), also known as monocyte chemoattractant protein 1 (MCP-1). CCL2 is expressed by both stromal cells and tumor cells (13) and is associated with poor prognosis in breast cancer (14, 15). Through recruitment of CCR2-expressing monocytes, CCL2 has been shown to promote pulmonary metastasis in mouse models of breast cancer (16). Activation of the CCL2–CCR2 axis promotes CCL3 production from macrophages, enhancing metastatic seeding of breast cancer cells (17). CCL5, also known as Regulated upon Activation, Normal T Cell Expressed and Secreted (RANTES), is another well-known factor that recruits TAMs to the breast tumor. CCL5 is expressed by malignant epithelial cells in breast carcinoma and is associated with advanced disease progression (18–20). Macrophages express high levels of its receptor (CCR5) and respond to CCL5 produced by tumor cells by infiltrating to the TME (21, 22). Importantly, CCL5 has been reported to alter the functionality of TAMs toward a tumor-promoting phenotype in colorectal cancer (23).

Several other factors produced by tumor cells help recruit macrophages. Colony-stimulating factor 1 (CSF-1), also known as macrophage colony-stimulating factor (M-CSF), and granulocyte-macrophage colony-stimulating factor (GM-CSF) are other tumor-derived factors produced by breast cancer cells (12, 24, 25). CSF-1 expression is associated with poor prognosis and increased infiltration of CSF-1 receptor (CSF-1R)-expressing macrophages in mouse models of breast cancer (26, 27). Tissue factors important for angiogenesis, such as vascular permeability factor (VPF), are also known to attract monocytes to tumors (28). In line with this, increased VEGF expression in tumor cells correlates with macrophage infiltration in human breast cancer (29). Hypoxia is another well-described factor that alters the activation and accumulation of macrophages in hypoxic regions and prevents migration out of these regions (30–32). Recruitment of TAMs into hypoxic niches occurs in a hypoxia-induced Semaphorin 3A (Sema3A)-dependent manner through VEGF-R1 phosphorylation in a mouse model of breast cancer (33). VEGF signaling and hypoxia also play a crucial role in angiogenesis, an important hallmark of cancer, providing links between angiogenesis and TAMs (34).

To further add complexity to TAMs, there are at least three lineages of macrophages that arise at different stages of development and persist into adulthood (35, 36). Each tissue in the body is composed of 5–20% tissue-resident macrophages, which are yolk sac-derived and seeded during embryogenesis. During homeostatic adaptations, such as tumorigenesis, macrophages of different phenotypes can be recruited from the monocyte reservoirs of blood, spleen, and bone marrow (37) and from resident progenitors or through local proliferation (38, 39). One report documented the loss of resident macrophages and a concomitant increase in monocyte-derived TAMs in a breast cancer model (40). Tissue macrophages have vastly different transcriptional profiles between tissues, suggesting that the macrophages at metastatic sites may differ from the primary tumor site and may need to be targeted differently (36). It is currently unknown how the ontogeny of TAMs influences primary and metastatic breast cancer and the extent to which TAM origin is important for clinical outcome.

### Differentiation and Maturation of TAMs in Breast Tumors

TAM differentiation and polarization is regulated by the tumor microenvironment (TME) and results in a heterogeneous population of cells, but the subset diversity is only just beginning to be understood. Tumors are generally poorly vascularized and lack nutrients, causing recruited monocytes to differentiate into mature wound-repairing macrophages. To an infiltrating monocyte, the tumor is a “wound” that needs repair, and therefore, TAMs polarize to an M2-like phenotype driven by CSF-1, IL-3, IL-4, IL-10, and TGF-β (Figure 2, step 2) (41, 42). TAMs subsequently show pro-tumor functions by promoting tumor cell survival, proliferation, angiogenesis, and dissemination (42–47). Whether or not differentiated macrophages are able to undergo M1/M2 repolarization after adopting a mature phenotype *in vivo* is still under debate, or if instead new monocytes recruited to the TME acquire their phenotype and remain in that state (41, 48–50). At least e*x vivo* M1-like human macrophages can be repolarized to M2-like macrophages upon exposure to M2 cytokines (51) and that M2-like macrophages are reprogrammed to express M1-like genes following exposure to TLR ligands or IFN-γ (52, 53).

## Pro-Tumor Functions of Macrophages in Breast Tumors

### Promotion of Tumorigenesis

The contribution of TAMs to cancer progression and outcomes is multifaceted due to their wide spectrum of phenotypes. *In vitro* and *in vivo* studies have shown that macrophages are involved in a feedback loop between tumor cells at invasive fronts of breast tumors, where macrophages provide factors that enhance tumorigenesis. TAMs can directly promote the proliferation of tumor cells by secreting growth factors and inflammatory mediators such as CCL2, IL-1α, IL-6, and TNF-α (Figure 2, step 3) (54). Importantly, TNF-α secreted by TAMs leads to the activation of NF-κB in tumor cells, preventing tumor cell death and enhancing tumor cell invasion (55). In that regard, tumor cells recruit and activate M2-like macrophages, which then produce M2-related cytokines, such as CCL18, which causes breast cancer cells to elongate, lose contact inhibition, and increase vimentin expression. In a humanized murine model, anti-GM-CSF and anti-CCL18 both reversed the epithelial–mesenchymal transition (EMT) state of cancer cells, inhibiting metastasis. This concept was further confirmed with human breast tumors, revealing that high GM-CSF expression significantly corresponds to CCL18-expressing macrophages, cancer cell EMT and metastasis, and poor clinical outcome (56).

### Facilitation of Angiogenesis

Angiogenesis is a complex, multistep process of forming new vasculature that facilitates tumor growth and progression (31). Macrophages are a major contributor of angiogenesis through the secretion of angiogenic cytokines (Figure 2, step 4) (57). TAMs secrete a key angiogenic cytokine, VEGF-A, directly, or indirectly by secreting matrix metalloproteinase 9 (MMP9) that activates latent forms of VEGF-A (29, 58, 59). TAMs also produce other pro-angiogenic factors from the fibroblast growth family that includes TGF-α, TGF-β, EGF, and PDGF (12, 60). Further supporting the role of macrophages in tumor angiogenesis, studies have shown that increased infiltration of TAMs promote angiogenesis whereas TAM depletion inhibits the angiogenic switch (61, 62). TAMs also contribute to angiogenesis by producing CCL18, which promotes angiogenesis both *in vitro* and *in vivo* (63). In addition to angiogenesis, CCL18 production from TAMs has also been associated with tumor invasiveness and metastasis in breast cancer (64). Furthermore, a subset of angiopoietin receptor Tie2-expressing monocytes, also known as TEMs, promote tumor angiogenesis (65, 66). In murine models, TEMs were initially identified as leukocytes expressing Tie2, CD11b, and CD45 in the peripheral blood and Tie2, CD11b, and Sca-1 in mammary tumors (65, 67). Additionally, TEMs also show a unique surface marker profile consisting of Tlr4, Mrc1, Il4ra, and CD163, which differentiate them from TAMs in murine mammary tumors (68). In humans, TEMs express CD45, CD11b, CD11c, CCR5, CD33, M-CSF-1R, and CD13 but lack expression of CD62L and CCR2 in circulating blood. Interestingly, TEMs from tumors of primary invasive breast carcinoma patients display different markers associated with antigen presentation, such as HLA-DR, CD80, CD86, and CD1a in addition to CD14, Tie-2, and VEGFR-1, which suggests their role in tumor-specific immune responses (69, 70). TEMs are also capable of secreting large amounts of IL-10 and VEGF, promoting a pro-angiogenic and immunosuppressive environment that inhibits tumor-specific T cell responses in breast tumors (70).

### Promotion of Metastasis

TAMs play a major role in tumor progression and metastasis by producing various matrix proteolytic enzymes including matrix metalloproteases (MMPs) and urokinase (uPA; Figure 2, step 5) (71, 72). This allows for the local invasion and attachment of cancer cells to other adjacent epithelial cells and the extracellular matrix (ECM) through the formation of adherens junctions. Next, the multistep process of invasion-metastasis occurs with intravasation of cancer cells to blood and lymphatic vessels, escape of cancer cells to distant tissues, and, finally, the growth of small lesions in tumors (34, 73). Direct visualization of macrophages using intravital multiphoton imaging showed that perivascular macrophages are important for the intravasation of cancer cells in the mammary tumor (74). In line with this, the genetic ablation of CSF-1 reduced macrophage density and slowed tumor progression and metastasis in the MMTV-PyMT model of breast cancer (75). CXCL1 secretion from TAMs is known to promote breast cancer migration and epithelial–mesenchymal transition in both mouse and human breast cells (76). Furthermore, a study showed that macrophages were required for breast tumor metastases, and Ets-2 transcription factor in TAMs was important for promoting angiogenesis and growth of both primary tumors and lung metastases (77). In another study, macrophage depletion using clodronate-encapsulated liposomes in an ovarian tumor reduced metastasis in ovarian cancer (78). In the MMTV-PyMT mouse model of breast cancer, TAMs show high levels of cathepsin protease activity, which promotes tumor invasion and angiogenesis (79). It is also reported that IL-4 released from tumor cells and T cells induces cathepsin protease activity in TAMs to promote tumor invasion (79).

Importantly, tissue-resident macrophages at metastatic sites may vary greatly. For example, hypoxic primary breast tumor environments release lysyl oxidase (LOX), triggering NFACTc1-driven osteoclastogenesis, which increases bone resorption and thereby creates a metastatic niche for circulating tumor cells. Bisphosphonate treatment inhibits bone osteoclasts, which could be a potential combination therapeutic for patients with high-LOX primary breast tumors (80). The ability for therapeutics to reach sites of metastasis should also be considered when targeting TAMs. For example, in a murine breast cancer model, local pulmonary administration of CSF-1R inhibition allowed the drug to overcome lung physiological barriers of administration better than oral administration, significantly enhancing the M1/M2 ratio at a much lower dose. Therefore, orally administered medications may not effectively reach the lung-resident macrophages due to mucous layers, enzyme degradation, or mucociliary clearance; therefore, locally administered routes specific to lung metastatic sites should be considered (81).

### Non-productive Antigen Presentation

Detrimental to the TME, protumor TAMs can become less efficient antigen-presenting cells, subsequently causing these cells to produce lower levels of pro-inflammatory cytokines and become less tumoricidal or able to activate T cells (82–84). TAMs that express pro-tumor features such as the IL-4R and arginase activity express low levels of MHCII (85). Subsets of TAMs characterized by lower expression of MHCII have been shown to be less effective in antigen presentation (85–87). In general, macrophages are less efficient in processing internalized antigens compared to conventional DCs, and macrophages in tumors appear to be greatly restricted in their antigen-presenting capacity (85, 86). Importantly, IL-10 derived from TAMs can also inhibit DC antigen presentation, further dampening tumor immunity (Figure 2, step 6) (88).

### Suppression of T Cell Function

TAMs release anti-inflammatory cytokines that promote an immune-suppressive tumor microenvironment through the recruitment of Tregs, which suppresses effector T cell activation (Figure 2, step 7). For example, CCL22 and CCL20 secreted by TAMs induce an immunosuppressive microenvironment by recruitment of Tregs in ovarian and colorectal cancer (89, 90). In addition, TAM-derived chemokines CCL17, CCL18, CCL22, IL-4, IL-10, TGF-β, and prostaglandin-E2 (PGE2) have all been well-characterized in their ability to suppress T cell functions (Figure 2, step 8) (91–97). As a third way to inhibit T cell function, blocking B7-H4 on macrophages improves macrophage-mediated T cell activation (98), and at the heart of T cell activation as it relates to immunotherapy, TAMs induce immunosuppression through the expression of ligands that bind to PD-1 and CTLA-4 molecules on T cells, which inhibits T cell activation (Figure 2, step 9) (99).

## Macrophage Metabolism as a Barrier to Anti-Tumor Immune Responses

Macrophage metabolism is a critical feature of the macrophage phenotype. Previous work has revealed that M2-like macrophages have a distinct metabolic profile compared to M1-like macrophages (100). *In vitro*, the metabolic profile of macrophages activated with IL-4 (M2-like stimuli) is distinct from those activated with lipopolysaccharide (LPS) + IFN-γ (M1-like stimuli) (100). Studies have revealed that IL-4-stimulated macrophages depend on fatty acid oxidation (FAO) known as β-oxidation (101) and glutamine metabolism for the production of key metabolic intermediates such as α-ketoglutarate and succinate (100), to fuel the oxidative tricarboxylic acid cycle (TCA) cycle. IL-4 activates glutamine catabolism in macrophages, and interestingly, glutamine deprivation or inhibition of N-glycosylation decreased M2 polarization and production of the chemokine CCL22 (100). However, macrophages stimulated with LPS and IFN-γ utilize glycolysis and fatty acid synthesis (FAS) (102). Some early work has revealed that enhanced uptake of glucose through the induction of glucose transporter 1 (GLUT1) is a key feature of LPS-activated macrophages (103). Furthermore, A[1,2-^13^C2] glucose tracer-based metabolomics approach, coupled with mass isotopomer distribution analysis of the newly formed metabolites, revealed that stimulated macrophages are highly glycolytic cells. The expression of 6-phosphofructo-2-kinase/fructose-2,6-bisphosphatase (PFK2), which regulates fructose-2,6-bisphosphate concentration and the glycolytic flux, was found to be the key molecular switch between the two phenotypic states and was independent of hypoxia-inducible factor 1α (HIF-1α) activation (104).

Metabolic regulation is a major difference between the extreme states of macrophage polarization and related to their downstream effector function. M1 macrophages preferentially depend on glycolysis, whereas M2 macrophages facilitates ATP production through the oxidative tricarboxylic acid cycle (TCA) coupled with oxidative phosphorylation (OXPHOS) (105–107). Previous work shows that M1 macrophages heavily depend on glycolysis to fulfill their energy demands; however, glycolysis only generates two ATP molecules per unit of glucose whereas the TCA cycle and OXPHOS generates 36 ATP molecules (108). It is speculated that M1 macrophages produce enough energy and intermediate metabolites for quick execution of effector functions like cytokine production and microbial killing (109, 110). Indeed, the metabolic differences between M1 and M2 macrophages are critical for their effector function. M1 and M2 macrophages display major differences in L-arginine metabolism via nitric oxide synthase (NOS) or arginase, respectively, which ultimately impact their functional outcome. NOS2 is activated through proinflammatory cytokines such as IFNγ, TNFα, IL-1β, and LPS (111) and has been well-characterized to metabolize L-arginine to L-citrulline and cytotoxic nitric oxide (NO; a pro-inflammatory mediator responsible for anti-tumor immune response) (112, 113). The expression of arginase is induced by Th2 cytokines such as IL-4 and IL-13 that activate the signal transducer and activator of transcription 6 (STAT6), which acts with STAT3 and CCAAT/enhancer binding protein β (C/EBPβ) to bind to the arginase 1 enhancer locus. Additionally, GM-CSF, TGFβ, prostaglandin E2 (PGE2), cyclic adenosine monophosphate (cAMP), and TLR agonists can induce the expression of arginase 1 (111). In M2 macrophages, arginase catalyzes L-arginine to urea and L-ornithine (113). In M2 (wound repairing) macrophages, ornithine production promotes cell proliferation and repairs tissue damage through generation of polyamines and collagen, which are important for wound repair, but highly immunosuppressive in the TME (114–117) and involved in mediating TME remodeling (118). Recent studies have challenged this dichotomy of macrophage metabolism. A transcriptomic and metabolomics-based analysis of macrophages stimulated either with LPS or Pam3CysSK4 [P3C; a toll-like receptor (TLR) 2 agonist] revealed divergent metabolic responses such as TCA, OXPHOS, and lipid metabolism pathways between both stimuli, which regulated cytokine production and phagocytosis. Unlike LPS, the TLR2 ligand P3C induced a complex metabolic rewiring by upregulating both glycolysis and OXPHOS (119).

### The Role of the TME on Mediating TAM Metabolism

The intricate communication between TAMs and the TME has been well-studied (36, 120–123), and the metabolic interactions between TAMs and cancer cells have been recently reviewed (124). There are growing efforts to understand the crosstalk between metabolic pathways, their metabolites, and the intracellular signaling in macrophages that in turn affects the epigenetic and transcriptional profile, which ultimately controls macrophage plasticity (100, 105, 125–127). One recent study showed that lactic acid production from tumor cells was an essential component in driving the M2-like polarization of TAMs by upregulating ARG1 and vascular endothelial growth factor A (VEGFA), with the effect of lactic acid being dependent on HIF-1α (128). Another study showed that lactate released from cancer cells acts as ligand for G protein-coupled receptor 132 (Gpr132, a pH sensor) that converts macrophages to an immunosuppressive M2-like phenotype. Importantly, genetic deletion of Gpr132 impaired M2-like macrophage phenotype with reduced tumor burden and metastasis in a preclinical model of breast cancer (129). In line with this, in areas of hypoxia, TAMs upregulate HIF-1α, which enables migration and survival (130), suggesting a feedback loop and signaling between tumor cells and TAMs that regulate their metabolic phenotype. Tumor-derived lactic acid has also been shown to induce the activation of the angiopoietin receptors (Tie1 and Tie2) and AXL receptors on TAMs. These receptors have been associated with pro-angiogenic and immunosuppressive phenotypes (131, 132). Similarly, macrophages treated with conditioned media from thyroid cancer cells underwent functional reprogramming. This effect was partly mediated by tumor cell-derived lactate that induced upregulation of cytokine production through an AKT1/mTOR-dependent increase in aerobic glycolysis. Immunohistochemistry (IHC) analysis confirmed the increase in glycolytic enzymes and lactate receptor in TAMs from human tumors (133).

Tumor cell-derived long-chain fatty acids including oleic acid have been shown to reprogram mitochondrial respiration in TAMs and subsequently induce polarization to an immune-suppressive phenotype through activation of the mTORC signaling pathway (134). In another study, tumor cell-derived succinate resulted in TAM polarization toward a pro-tumor phenotype through a succinate receptor (SUCNR1) and was dependent on the PI3K/HIF1α signaling pathway that resulted in enhanced metastasis (135). Similarly, prostaglandin E2 (PGE2), a prostanoid lipid synthesized by tumor cells, has been reported to polarize TAMs toward a pro-tumor phenotype through the cyclic AMP-responsive element binding (CREB) pathway (136, 137). These studies indicate that there is an intricate relationship between the factors secreted by the TME that dictates the outcome of macrophage phenotype and function. Additional studies to evaluate the differences of how the TME at different metastatic sites regulates TAM metabolism are warranted. An in-depth understanding of the crosstalk between TAMs and TME will be critical to design therapeutic strategies to target TAMs at both the primary and metastatic sites.

## Clinical Significance of Tams in Breast Tumors

TAM infiltration is associated with poor prognosis, in a variety of cancers, including breast cancer (61, 92, 138, 139). This finding has been corroborated in a meta-analysis where TAM density significantly correlated with poor survival in patients with breast cancer (140). In an analysis of 11,000 breast tumors, the immune cell type that correlated most significantly with poor clinical outcome in estrogen receptor-positive (ER+) breast tumors was the presence of TAMs (141, 142). Clinically, the presence of TAMs is associated with metastasis (44) and poor survival (45–47) and has been shown to induce endocrine therapy resistance in ER+ breast cancer cells *in vitro* and *in vivo* through NF-κB- and IL-6-dependent signaling pathways (143). Importantly, a higher fraction of M1-like TAMs in ER+ breast cancer correlated with a higher pathological complete response (pCR) rate as well as prolonged disease-free survival (DFS) and overall survival (OS) (142). In a cohort of 40 HER2+ breast cancer patients who received trastuzumab, an anti-HER2 therapy, a high number of M1-like macrophages (iNOS+) were significantly associated with improved survival whereas high expression of M2-like macrophages (CD163+) were associated with worse prognosis (144). Additionally, high numbers of iNOS+ M1-like macrophages combined with high numbers of CD8+ T cells were significantly associated with improved survival, and this combined marker predicted a patient's ability to remain progression-free without trastuzumab after initially responding to therapy (144). This is in line with other reports demonstrating low TAM and high CD8+ T cell populations are associated with better recurrence-free survival in patients with invasive breast cancer (145).

### Relative Numbers of Macrophages Across Breast Tumor Subsets

Evidence for differential TAM regulation between breast tumor subsets comes from ER+ and triple-negative breast cancer (TNBC) cell lines co-cultured with macrophages. Tumor-induced phenotypic drift toward M2-like phenotype has been shown in differentiating bone marrow-derived macrophages (BMDMs) in breast cancer cell-conditioned media (146). Additionally, macrophages exposed to a TNBC cell line upregulated CCL2, reinforcing the concept that macrophage infiltration is a vicious feedback loop. In contrast, ER+ breast cancer cells co-cultured with macrophages were more broadly pro-inflammatory, secreting CXCL10, IL-2RA, and IL-3 (147). Furthermore, macrophage infiltration by breast cancer subtype has been explored using gene expression data with adjacent normal tissue as a baseline, which reported lower macrophage scores in ER+ tumors, especially ER+/HER2– subsets, in comparison to ER– tumors (148). A recent study examined immune-rich ER+ and immune-rich TNBC tumors based on microarray expression scores and compared relative fractions of immune cells. Although TNBC had a higher overall macrophage count, ER+ tumors had a higher relative percentage of M2-like macrophages and upregulation of TGF-β expression, which are both indicators of poor prognosis (149). TGF-β and IL-4 have been shown to upregulate YKL-39, a chitinase-like protein secreted by TAMs, which facilitates monocyte recruitment and angiogenesis. Importantly, YKL-39 expression levels were six times higher in patients with metastases than without metastases after neoadjuvant chemotherapy (NAC), independent of tumor stage or grade (150). YKL-39 has also been shown to be elevated in estrogen receptor 1 (ESR1) mutant metastatic breast cancer (151) and has been shown to be regulated by the androgen receptor in prostate cancer (152). These differences highlight unique targeted therapies, such as targeting the TGF-β pathway or reprogramming M2-like macrophages into M1-like macrophages, as having high potential, especially in ER-positive breast cancer.

**Figure 3:**
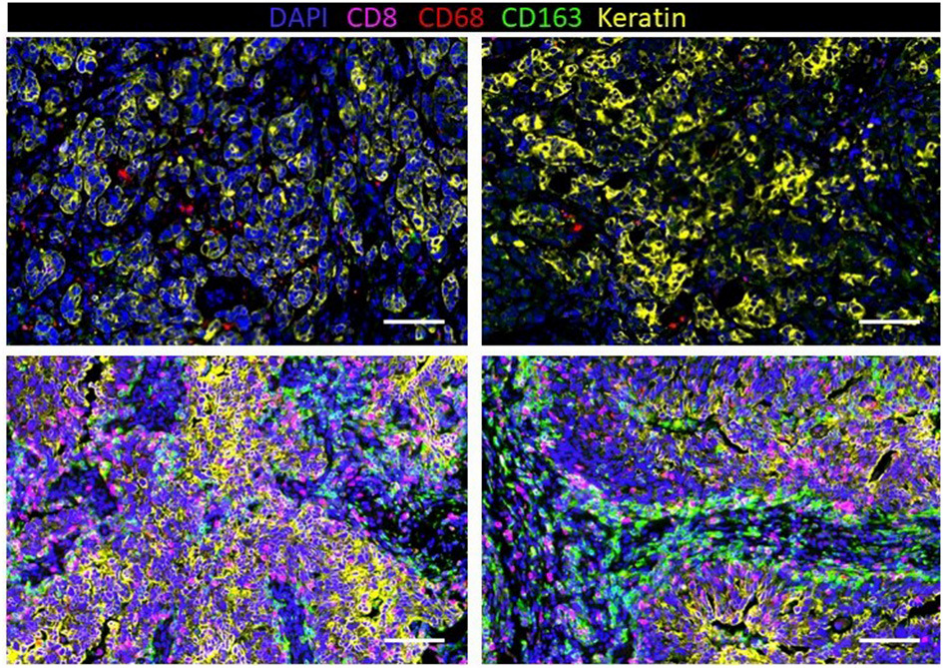
Infiltration of macrophages (CD68 and CD163) and T cells (CD8) in human triple-negative breast cancer (TNBC) with a low density of immune cells (top row) and high density (bottom row).

Immunohistochemistry (IHC) has revealed that TNBC has significantly more tumor-infiltrating T cells and macrophages compared to non-TNBCs (153). To further understand the extent of macrophage infiltration in TNBC, immune infiltrates in 41 TNBC tumors were characterized using multiplexed ion beam imaging by time of flight (MIBI-TOF) (154). The fraction of infiltrating immune cells ranged widely from 1 to 91% of cells in the analysis fields selected from each tumor. On average, macrophages were among the most abundant immune cell in the tumor region, accounting for 25% of the immune population. Other immune populations also significantly contributed to the immune landscape, including the following: CD8^+^ (19%), CD4^+^ (15%), regulatory T cell (1%), B-cells (11%), and NK cells (<1%). However, these numbers varied greatly between patients. In fact, macrophages comprised as high as 100% of the immune population in tumors with low immune infiltration or as low as 5% in more immunologically abundant TNBC tumors. Our group has recently investigated macrophage abundance in TNBC using multiplex cyclic immunofluorescence (CyCIF) (155–158). We also found that TAM infiltration can vary greatly between tumors (Figure 3). In addition, we recently reported that TNBC harboring mutations in the breast cancer gene 1 (BRCA1) has a nearly 10-fold increase of TAMs compared to *BRCA1*-wildtype TNBC (159). This work highlights that tumor cell intrinsic mutations, such as BRCA mutations, may play a key role in regulating the TME. Indeed, the current and other studies have shown that BRCA-deficient cancer cells have an increase in cytosolic DNA leading to STING pathway activation and secretion of type 1 interferon-related cytokines such as CXCL9 and CXCL10 that can recruit immune cells, including macrophages, to the tumor (160, 161). Another work has confirmed an increase in CD68+ macrophages in TNBC compared to other subsets, and interestingly, breast tumors with high frequency of c-Myb-positive cells, identified through mRNA levels in breast cancer patients from public datasets, were correlated with a lower density of CD68+ macrophages, which was also found within the molecular subtypes (162).

### Relative Numbers of Macrophages in Breast Cancer Progression

Not only does macrophage abundance vary between breast cancer subtypes but also with the stage of cancer progression. In preclinical models, macrophages have been shown to orchestrate early breast cancer dissemination and metastasis in a mouse model of HER2+ breast cancer, where CCL2 produced by both cancer cells and myeloid cells recruited CD206+/Tie2+ macrophages to propagate the disease (163). Similar results were seen in the MMTV-PyMT murine model of luminal B breast cancer, where blocking CCL2 prevented recruitment of TAMs to breast tumors, reduced metastasis, and prolonged the survival of mice (16). Other preclinical studies have shown a significant correlation between CSF-1 and breast cancer metastasis using the MMTV-PyMT model (26).

Compared to normal breast tissue, macrophage numbers are significantly higher in ductal carcinoma *in situ* (DCIS) and remain higher through progression to invasive ductal carcinoma (IDC) (164). TAMs from human breast cancer have been shown to have a distinct transcriptomic signature from macrophages in healthy breast tissue, which is enriched in more aggressive forms of breast cancer (165). Additionally, CD68+ macrophages (166, 167), especially those defined by authors as M2 macrophages (CD68+PCNA+), have been shown to be elevated in high-grade compared to low-grade DCIS (167). Recent work by Gil Del Alcazar et al. compared immune cell infiltration in HER2+ and TNBC as a function of disease progression by comparing DCIS to IDCs (168). IDC had higher numbers of macrophages compared to DCIS. Interestingly, in DCIS, higher fractions of CD8+ T cells were associated with a significantly higher frequency of macrophages. Gene expression profiling revealed that Th1 and Th2 cells were enriched in HER2+ IDCs, while TNBC IDCs had enrichment of Th17 cells and T regulatory cells (Tregs). For TNBC tumors, but not HER2+ tumors, the transition from DCIS to IDCs correlated with an increased number of TILs, but fewer were in the activated state, indicative of T cell exhaustion in advanced stages of TNBC. Macrophages were not examined in this context, indicating that further characterization is needed to fully understand how macrophages participate in different stages of cancer progression.

### Location of Macrophages in Breast Tumors

In addition to breast cancer subtype and disease state, the location of macrophages within the TME may be a predictor of their function and correlate with clinical outcome. However, the field has not yet come to a consensus on the extent of region-specific TAM behavior as a prognostic factor. Generally, macrophages in the stroma are associated with angiogenesis, immunosuppression, and cancer cell migration. In contrast, macrophages in the cancer nest tend to be more heterogeneous across cancer types and are correlated with reduced overall survival (OS) and recurrence-free survival (RFS) in breast cancer (169). Gwak et al. found stromal, intratumoral, and total TAMs to all have similar prognostic value, while Merdeck et al. reported stromal TAMs, but not tumor nest TAMs, are significantly correlated with tumor size and high tumor grade (45, 170). Recently, CD68 and CD163 were evaluated in both tumor nests and tumor stroma in 60 specimens of invasive breast cancer. High numbers of CD68+ TAMs in the tumor stroma were significantly associated with larger tumor size and positive nodal metastasis, whereas high numbers of CD163+ TAMs in the tumor stroma were significantly associated with positive vascular invasion, nodal metastasis, and molecular subtypes (171). Macrophage behavior may be modulated by breast cancer subtype and TME location, which in turn may regulate tumor behavior with stimulatory and inhibitory signals. For example, in HER2+ and basal-like subtypes, macrophages concentrate in invasive fronts and use TGF-β signaling to thicken the extracellular matrix (ECM), contributing to breast tumor metastasis (172). Therefore, not only are macrophages shaped by their location within the TME but they also reciprocally influence the surrounding TME composition. The significance of histologic localization of TAMs and the degree of TAM infiltration add additional layers of complexity to targeting TAMs in breast tumors for anti-cancer therapy.

### Macrophage Characterization After Therapy

The characterization of TAMs before and after therapy may provide insight to how TAMs may change with treatment and play a role in drug resistance and metastasis. Increases in macrophages have been reported after chemotherapy in both preclinical and clinical settings (145). In a primarily ER+ patient cohort, neoadjuvant chemotherapy increased the percentage of CSF-1R+ macrophages (173). In line with these findings, Waks et al. examined changes in TAM populations after neoadjuvant chemotherapy in ER+ breast cancer, reporting an influx of CD68 macrophages with a larger proportion associating with an M2-like phenotype (174). These findings are in line with preclinical studies showing chemotherapy significantly increased F4/80+ macrophages in chemotherapy-sensitive tumors, but not chemotherapy-resistant tumors, indicating that macrophages are recruited to the site of chemotherapy-induced apoptosis and play a role in drug response (175). These changes in the TME could be implicated in mechanisms of chemotherapy resistance.

## Macrophages as a Barrier to Chemotherapy and Immunotherapy

While the heterogeneity of TAM populations is still being deconvoluted, clinical evidence suggests that macrophages can contribute to shortcomings of chemo- and hyphenate immuno-therapy. High TAM density has been shown to be an independent poor prognostic marker in breast cancer patients, especially for HR-positive breast cancer (170). Importantly, macrophages have been shown to contribute to reduced efficacy of chemotherapy. Shree et al. discovered that *in vitro* cathepsin-expressing BMDMs shield mammary cancer cells from paclitaxel-induced cell death. They further demonstrated that tumors from MMTV-PyMT mice treated with paclitaxel had increased TAM infiltration, and cathepsin inhibition in combination with paclitaxel increased long-term survival (176). Similarly, tamoxifen-resistant ER-positive and HER2-negative clinical samples had a higher density of CD163+ macrophage populations and increased expression of EGFR than tamoxifen-sensitive samples, which positively correlated with tumor size and metastasis (177). Furthermore, macrophages can disrupt T cell infiltration, which immunotherapies rely on to mediate efficacy. For example, in lung cancer patients, macrophage exclusion of CD8+ T cells from tumor nests correlated with poor response to anti-PD-1 therapy. When macrophages were depleted with anti-CSF-1R therapy, CD8 T cells successfully infiltrated the tumor to interact with malignant cells and delay tumor progression (178). Also, importantly, in preclinical breast cancer models, when macrophages are depleted using anti-CSF-1R therapy (145, 178) or their phenotype was converted to an anti-tumor phenotype (179), anti-PD-1 therapy induced potent anti-tumor immunity. This highlights the deleterious effects of TAMs in tumors and the importance of targeting both innate and adaptive immune cells to achieve the full potential of immunotherapy and a durable anti-cancer immune response.

### Targeting TAMs for Anti-Cancer Therapy

Both preclinical and clinical strategies to target the tumor-promoting functions of TAMs in cancer are being developed. These approaches have been reviewed in great detail and include inhibiting the recruitment of macrophages to tumors by blocking the CCL2–CCR2 or CCR5–CCL5 axes, depleting TAMs by blocking CSF-1 or CSF-1R; blocking macrophage “checkpoint inhibitors” such as CD47/SIRP1α, PD-1/PD-L1, MHCI/LILRB1, and CD24/Siglec-10; and suppressing macrophages' pro-tumor activity (inhibition of TGF-β or VEGF) (36, 180–184). Depletion or inhibition of macrophages using CCL2, CSF-1, and CSF-1R inhibitors has been shown to be effective against both mouse and human tumors (16, 36, 145, 185). Importantly, a recent study showed that CCL2 inhibition as a monotherapy led to more metastasis when the therapy was discontinued, which was driven in an IL-6- and VEGF-dependent manner (186). This study challenges the use of CCL2 as a monotherapy and highlights the need to understand the tumor microenvironment composition for successful anti-metastatic therapy.

CSF-1R is a promising target to address TAMs therapeutically, as high expression of CSF-1 or CSF-1R predicts cancer progression and mortality (187). Blockade of CSF-1R has been shown to decrease TAM infiltration, which subsequently results in the increase in CD8+ T cells and improves response to chemotherapy (95, 145). In a phase Ib study with advanced solid tumors, the combination of pexidartinib, a CSF-1R inhibitor, and paclitaxel was well-tolerated, and the combination showed reduced macrophage infiltration in the tumor microenvironment (188). However, in another phase I a/b study, emactuzumab, a monoclonal antibody against CSF-1R, showed reduction in immunosuppressive TAMs but did not demonstrate clinical benefit alone or in combination with paclitaxel (189). These studies suggest that a careful evaluation of the TME is important before deciding which patients would best benefit from CSF-1R therapy. Other caveats to anti-CSF-1R therapies include reports showing that inhibition of CSF-1R signaling can promote breast cancer metastasis (190).

To enhance anti-CSF-1R therapies, combining anti-CSF-1R with complementary chemotherapy and agents that enhance T cell function may markedly improve outcomes. In that regard, a recent study showed that addition of a CD40 agonist before anti-CSF-1R therapy induced a short-lived hyperactivated macrophage state that was enough to generate an effective T cell response in ICB-resistant tumors (191). Additionally, we have recently shown that CSF-1R inhibition leads to a significant reduction in TAMs and when combined with PARP inhibitor therapy results in an increase in overall survival, with some mice experiencing tumor-free survival for at least 1 year (159). Studies with CSF-1R signaling antagonists, combined with the drug paclitaxel or carboplatin, showed enhanced tumor control and reduced metastasis in preclinical models of breast cancer. Importantly, blockade of CSF-1 signaling also enhanced anti-tumor immunity and cytotoxic T cell infiltration to chemotherapy (145). The blockade of the CCR5–CCL5 axis, which decreased macrophage infiltration in tumors, is another exciting therapeutic target with ongoing clinical trials for breast cancer (192).

**Figure 4:**
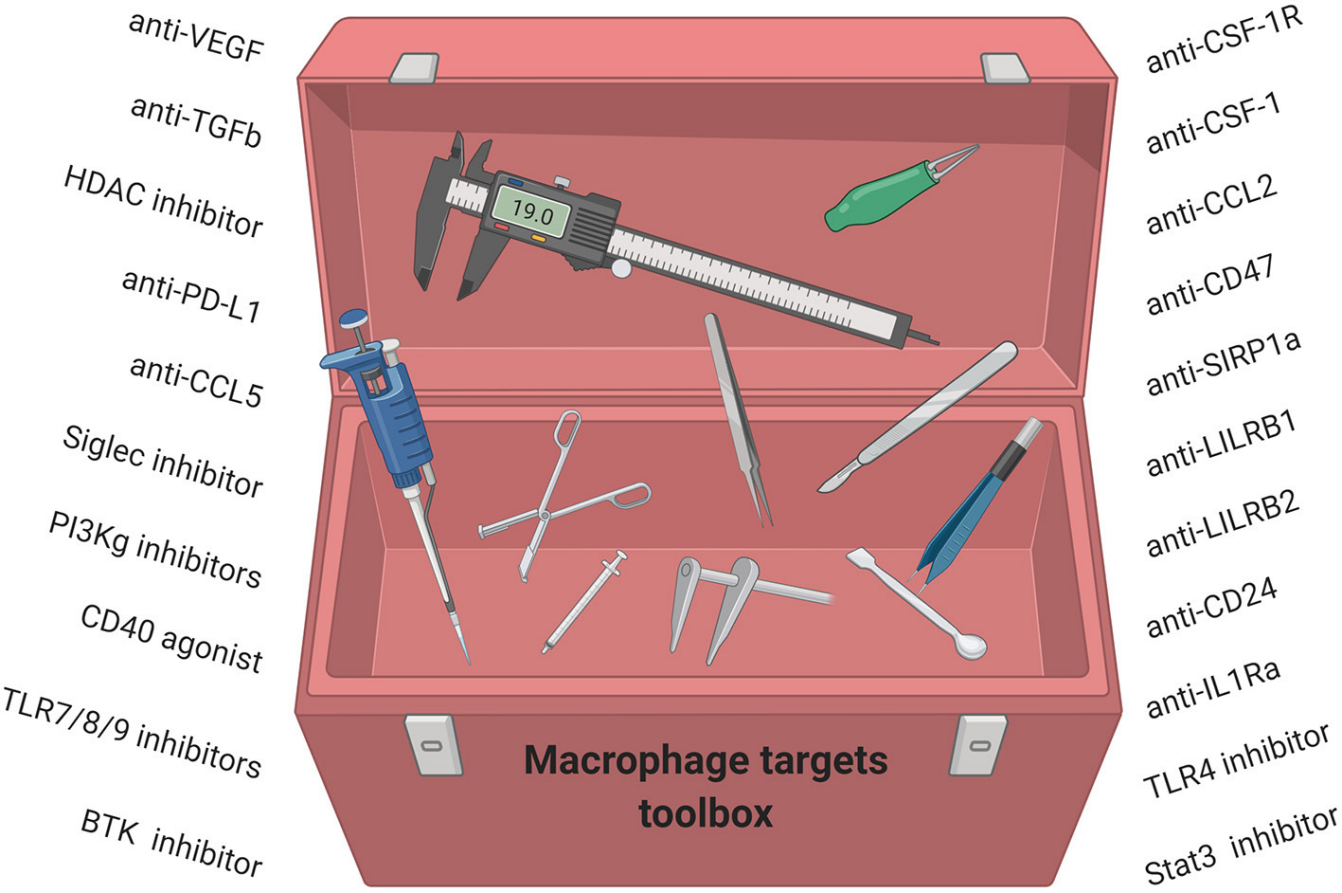
Macrophage-targeting strategies for anti-cancer therapy have started to fill our toolbox. We now need to understand how these compounds work, which subsets of TAMs they modulate, and which breast cancer patients will benefit.

An alternative strategy is to convert pro-tumor TAMs to an anti-tumor phenotype. CD40 agonists (193), PI3Kγ inhibitors (194), CD47 inhibitors (195), and a class IIa HDAC inhibitor (179) have all been shown to reduce primary and metastatic murine breast tumors (179) and have emerged as novel modalities to convert TAMs to anti-tumor macrophages. In addition, other strategies have been shown to convert TAMs to an M1 phenotype and include Bruton's tyrosine kinase (BTK) inhibitors (196), TLR agonists (197), STAT3 inhibitors (198), IL-1Ra inhibitors (199), and LILRB2 inhibitors (200) Taken together, strategies to deplete or inhibit suppressive TAM functions or activate anti-tumor TAMs combined with chemotherapy and/or immunotherapy may have a great potential for the treatment of breast cancer patients. However, while many of these compounds in preclinical and clinical development are now filling our toolbox with TAM-targeting strategies, it will likely be necessary to further elucidate the complexity of TAM subsets including their ontogeny and phenotype for successful therapeutic targeting (Figure 4).

### Therapeutic Targeting of TAM Metabolism for Anti-Cancer Therapy

The metabolic programming of TAMs is complex, and the underlying molecular mechanisms and crosstalk between tumor cells and stroma remain to be characterized. An in-depth analysis of these metabolic circuits may facilitate better appreciation for the functional fates of macrophages, including their pro- vs. anti-tumor phenotype. This important information would further support the clinical application of targeting TAM metabolism for anti-cancer therapy. There is some insight of the potential of this strategy from several recent publications that utilized other immune cell types including Tregs. Recently, Tregs were shown to activate the sterol regulatory element-binding protein 1 (SREBP1)-mediated fatty acid synthesis pathway in TAMs. SREBP1 induced M2-TAM metabolic fitness, mitochondrial integrity, and survival (201). Pharmacological inhibition of *de novo* fatty acid synthesis using a SREBP1 inhibitor, fatostatin, showed anti-tumor immunity when combined with ICB (anti-PD-1) in a B16 melanoma preclinical tumor model (201). Our group recently reported that PARP inhibition directly modulated macrophage metabolism by shunting glycolysis and inducing a dependence on lipid metabolism, which generated an immunosuppressive TME by inhibiting T cell function and thereby contributed to PARP inhibitor resistance (159). The use of fatostatin in combination with PARP inhibition and macrophage modulation significantly enhanced the overall survival of mice bearing brca1-deficeint TNBC (159). In line with our findings, inhibition of PARP induced upregulation of lipogenic genes by modulating the transcription factor specificity protein 1 (Sp1), which leads to the accumulation of lipid droplets in the liver (202). Similarly, a study suggested that genetic deletion as well as pharmacologic inhibition of PARP induced the expression of ATP-binding cassette transporters (ABCA1) and cholesterol efflux in macrophages (203). These studies highlight the role of both Tregs and PARP inhibitors in regulating macrophage lipid metabolism. Further molecular understanding on the mechanisms of how PARP inhibitors regulate TAM metabolism would provide future opportunity for promising therapeutic strategies.

In a preclinical syngeneic model of pancreatic ductal adenocarcinoma (PDAC), a TLR9 agonist, CpG oligodeoxynucleotide, induced a metabolic state that required fatty acid oxidation and shunting of TCA intermediates for *de novo* lipid biosynthesis. This shift in central carbon metabolism activated highly phagocytic macrophage that could overcome the CD47 “don't-eat-me” signals on tumor cells to mediate an antitumor response (204). Macrophages cultured with PDAC-conditioned media compared to normal pancreatic cells had higher levels of vascular network formation, enhanced metastatic potential, increased levels of EMT, and a pronounced glycolytic signature. Inhibiting hexokinase II (HK2) with 2-deoxyglucose (2DG) inhibited glycolysis and reversed the pro-tumor TAM phenotype, highlighting the therapeutic potential of modulating TAM metabolism for anti-cancer therapy (205). Molecular metabolic control of TAMs has been demonstrated *in vitro* by inhibiting glutamine synthetase (GS). In human monocytes, GS expression activates an M2-like phenotype, which is reversed through pharmacological inhibition of GS by methionine sulfoximine (MSO). Inhibition of GS resulted in production of succinate, a critical regulator of the pro-inflammatory response, and enhanced glucose flux through glycolysis. Importantly, in *ex vivo* studies, GS restored T cell recruitment. *In vivo*, genetic deletion of macrophage-specific GS reduced metastasis in a preclinical mouse model of lung cancer (206). Taken together, precisely targeting the metabolic rewiring of TAMs may re-educate their phenotype and may overcome TAM-associated immunosuppression.

## Beyond M1 And M2 Phenotypes and Next Steps

To date, characterizing the diversity of macrophage subsets has been difficult, as researchers have relied on a limited number of macrophage markers, and gene expression profiling has been done in bulk tissue or total macrophage populations, which preclude detection of unique subsets. Although gene expression data is high throughput, it inherently lacks the ability to determine spatial relationships, precise cellular function, or biochemical analysis at a single-cell level. Another consideration for leukocyte-containing samples is the canonical expression of RNase, which could potentially degrade RNA transcripts and interfere with single-cell analysis sample quality. Likewise, protein analysis should be used to support gene expression data, but is comparably tedious and low yield. The intracellular glycoprotein CD68 is widely used in clinical studies as a TAM marker, but it also detects other cell types such as some lymphoid and non-hematopoietic cells (207, 208) and does not identify TAM phenotype or functional status. The scavenger receptor CD163 has also been used to identify some TAM populations and has been shown to associate with early recurrence and reduced survival in breast cancer patients (209). CD68 and CD163 have been broadly used to show that high TAM density correlates with a worse clinical outcome in breast cancer but do not predict their functional phenotype (122, 140, 210). As high-resolution techniques uncover the heterogeneity and plasticity of macrophage populations, researchers will need to look beyond the binary M1/M2 nomenclature and incorporate an extensive panel of markers in their analysis. Indeed, TAMs have high plasticity and can express both M1-like and M2-like phenotypes simultaneously, creating a need for more nuanced categorizations of macrophages (169). Multiplex IF such as MIBI-TOF and CyCIF have the unique advantage of analyzing a relatively large number of proteins, while maintaining spatial context of the tumor. These technologies will be invaluable in the next era of TAM studies, in which distinct phenotypes will need to be pursued.

Immune-focused mass cytometry has shed light on the diversity of TAMs in breast cancer, where in a study of 144 breast cancer patients, 19 distinct subsets of myeloid cells were identified, which clustered into five broader categories (211). These clusters included CD14-expressing monocytes, early immigrant macrophages, tissue-resident macrophages, TAMs, and myeloid-derived suppressor cells, with each group containing several additional subsets of myeloid cells. The distribution of the 19 clusters was distinct regarding the location within the tumor microenvironment and the subtype of breast cancer. For example, ER– tumors had more PD-L1+ TAMs than ER+ tumors, while luminal B tumors contained a higher proportion of PD-L1+ TAM subsets than luminal A. Although the sample set was predominantly from ER+ tumors, the results expose high-resolution categorizations of macrophages that could shed light on current therapeutic barriers. This work highlights the complexity of macrophages and tumors and is the right step forward; however, it is yet to be understood what the functional significance of these subsets are for clinical outcomes (211). Assays that test the functional ability of macrophages are warranted including high-throughput efferocytosis assays or gene signatures that may predict enhanced efferocytosis. The abovementioned studies have contributed to understanding the immune profile related to macrophages in breast cancer subtypes using bulk and single-cell RNA-sequencing, single- and dual-color IHC, multiplex spatial analysis such as MIBI-TOF and CyCIF, as well as flow cytometry, which compile data with different perspectives and limitations. As researchers continue to assess the breast TME, it is critical to acknowledge the strengths and limitations of different methods regarding their resolution, accuracy, and ability to represent tumor heterogeneity.

TAMs play a major role in tumor progression and metastasis and promote a highly suppressive TME that may limit breast cancer therapy. Therefore, modulating TAMs in combination with chemo- and/or hyphenated immuno-therapy will be critical to achieve maximum tumor reduction and elimination. Thus, it is imperative to characterize TAM subsets and their location. In addition, differences among breast tumor subtypes and how TAMs change after therapy will be important to characterize, along with differences in tissue-resident macrophages at metastatic sites. To develop effective macrophage-targeting therapies for the treatment of breast cancer, it is critical to have a precise understanding of the unique macrophage populations in each subset of breast cancer, as well as their location within different regions of the tumor. In addition, when considering the variety of influences that macrophages have on the TME, it is apparent that the surrounding TME will also need to be considered. Therefore, it is crucial to assess the number of infiltrating macrophages, their phenotype and function, as well as their spatial location to other cells in the TME. Expanding beyond M1 and M2 macrophage nomenclature will enhance the field of cancer immunology by providing better understanding of the tumor environment for rationale design of immunotherapy strategies including future development of macrophage-targeting therapies (Figure 4).

## Author Contributions

All authors listed have made a substantial, direct and intellectual contribution to the work, and approved it for publication.

## Conflict of Interest

JG is a consultant for Glaxo-Smith Kline (GSK), Codagenix, Verseau, Kymera, and Array BioPharma and receives sponsored research support from GSK, Array BioPharma, and Eli Lilly. The remaining authors declare that the research was conducted in the absence of any commercial or financial relationships that could be construed as a potential conflict of interest.
